# Optical properties of artificial intraocular lenses and considerations for additive manufacturing

**DOI:** 10.3389/fmed.2025.1563766

**Published:** 2025-04-14

**Authors:** Bushra Brighesh, Noran Alsuliman, Ahed Nimer, Omnyah Albosaad, Batool Alokosh, Tala Alnaili, Abeer Syed, Michael R. Gardner

**Affiliations:** Department of Biomedical Engineering, College of Engineering, King Faisal University, Al Ahsa, Saudi Arabia

**Keywords:** intraocular lens, optical transmittance, optical scattering, additive manufacturing, cataracts

## Abstract

Cataracts, a leading cause of blindness in the world, are commonly treated by replacing the ocular lens with an artificial intraocular lens (IOL). The material and structure of the IOL are major factors in their efficacy, affecting, among other characteristics, the optics of the eye. In the recent research record, two optical properties have emerged as standardized characterization methods for IOL optics: (1) optical transmittance and (2) optical scattering. This mini review describes these two methods and collates data in such a way that comparisons may be drawn across four different IOL material types (PMMA, hydrophobic acrylic, hydrophilic acrylic, and silicone) and three IOL conditions (*in-vivo*, cadaver explant, and inventory control). Finally, the emerging field of additive manufacturing for IOL production is considered. Such technologies hold promise for optimizing IOLs for cataract patients. Researchers in additive manufacturing for IOL production may incorporate optical transmittance and optical scattering as standard characterization methods for 3D-printed IOLs developed by the broader IOL researcher community.

## 1 Introduction

As people age, the proteins of the ocular lens can undergo abnormal structural and chemical changes, pigmentation, and stiffening (1). These changes lead to areas of cloudiness in the ocular lens known as cataracts, which contribute to visual symptoms such as blurred vision and decreased contrast sensitivity. Left untreated, cataracts often lead to blindness. Almost 95 million people suffer from cataracts, the leading cause of preventable vision impairment worldwide (2).

Surgery is the primary treatment for cataracts (1), and the most common method of cataract surgery is an outpatient procedure in which a foldable artificial intraocular lens (IOL) is placed within the capsular bag to provide anatomic stability and mimic the refractive power of the ocular len (3–5).

The material and structure help determine the efficacy of IOLs. Important considerations for material selection include optical properties, bio-compatibility, transparency, and biomechanics. The IOL's structural design determines mechanical fit and can inform the surgical procedure itself, and the surface geometry—along with the refractive index—of the IOL help defines its refractive power (6).

Although cataract surgery is known to be one of the most successful surgeries in the world (7), emerging manufacturing processes such as additive manufacturing are creating an opportunity for improved outcomes and patient-centered care. Patient-specific ocular characteristics may inform preoperative considerations and optimize surgical outcomes (8). Beyond mechanical fit and bio-compatibility (9), IOL optical properties are key to optimizing IOL performance for improved vision.

This mini review paper examines two key optical properties of IOLs: (1) optical transmittance and (2) optical scattering. We compare these well-studied properties across various IOL types and studies. While the optical properties of IOLs explanted due to failure were reported in many studies, the mechanisms of failure were often unreported and, in many cases, may have been due to factors exogenous to the IOL itself. Instead of including explanted failed IOLs to described longitudinal performance of IOLs, we have opted to limit inclusion to pre-implanted controls or healthy functioning IOLs explanted from cadavers when the data is available.

Finally, we review emerging additive manufacturing technologies for IOL production. This mini review aims to provide a summary of key optical characteristics of IOLs for consideration in emerging IOL manufacturing approaches.

## 2 Optical transmittance

Optical transmittance of the ocular lens refers to the amount of light that passes through the lens to be focused onto the retina. As with other optical materials, the ocular len's transmittance is a function of many parameters, including the wavelength of light. In the healthy human eye, optical transmittance is optimized for visible wavelengths, to which the rods and cones of the retina are sensitive. The lens is an optical filter that controls the amount and type of radiation that passes through it (10). In their canonical study, Boettner and Wolter (11) reported the transmission of light at 80% from 360 nm, increasing rapidly to 90% between 450 nm and 540 nm, depending on age (older patients have a slower increase). The lens' high transmittance continues out to 1,400 nm, with typical water bands at 980, 1,200, and 1,430 nm. The lens' transmittance reduces with age due to changes in the lens' protein structures, commonly associated with cataracts (10, 12).

To replace the cataract lens with an artificial IOL, the transmission of the material should be carefully considered. Many such studies have been performed for different kinds of IOLs. In this review, 10 studies were selected for the discussion of optical transmittance. The reports of IOL optical transmission included in these publications were reported for a diverse set of research objectives and often included measurements of both explanted IOLs (i.e., removed from patient due to failure) and control IOLs from inventory. Because IOL failure is multifactorial and the reasons for failure were not consistent across studies reporting optical transmission, only control/inventory IOLs (i.e., pre-implantation) transmission values are reported in this section.

Importantly, each of the studies was performed using spectrophotometry. The selected studies include optical transmittance evaluations of the most common commercially available IOL materials (7, 13): (1) poly(methyl methacrylate) (PMMA), (2) hydrophobic acrylic, (3) hydrophilic acrylic, and (4) silicone. Three studies examined the differences between hydrophobic acrylic IOLs with (yellow) and without (clear) blue-light filtering chromophore (14–16), and one study evaluated the transmittance of PMMA vs PMMA copolymers (13, 17). Where data were available or data extraction from plots was possible, the transmittance values from each study are listed over a selection of visible wavelengths (450, 600, and 750 nm). Additionally, overall transmittance values are listed when reported. The exact method for calculating overall transmittance is unreported in most reviewed publications, but in most cases seems to be an average transmittance level (%) from around 300 nm extending to 800 nm. See Table 1.

**Table 1:** A review of optical transmittance values for different IOL materials.

		**Transmittance (%)**			
**References**	**Material**	***450 nm***	***600 nm***	***750 nm***	***Overall***
Wang et al. (13)	PMMA	93.1	93.8	94.5	-
	0.05 MA POSS-PMMA	99.1	99.1	99.3	-
	0.10 MA POSS-PMMA	99.1	99.3	99.4	-
	0.25 MA POSS-PMMA	99.2	99.2	99.2	-
	0.50 MA POSS-PMMA	99.4	99.5	99.6	-
Wang et al. (17)	PMMA	93.1	93.9	94.5	-
	0.01 allyl POSS+PMMA	91.4	92.3	93.1	-
	0.02 allyl POSS+PMMA	90.5	91.0	91.7	-
Michelson et al. (19)	PMMA	99.7	99.6	99.8	98.8
	Hydrophilic Acrylic	98.8	99.4	99.7	98.0
	Silicone	94.6	99.0	99.2	97.7
Werner et al. (18)	PMMA	-	-	-	98.8
	Hydrophilic Acrylic	-	-	-	97.9
	Silicone	-	-	-	97.7
Barra et al. (69)	Hydrophilic Acrylic	98.0	98.7	99.1	98.2
Matsushima et al. (70)	Hydrophobic Acrylic	97.5	98.9	99.6	89.0
Yoshida et al. (71)	Hydrophobic Acrylic	94.1	95.6	96.1	85.7
Werner et al. (14)	Hydrophobic Clear Acrylic	98.4	99.2	99.3	96.9
	Hydrophobic Yellow Acrylic	54.2	98.0	98.3	83.2
Bhattacharjee et al. (15)	Hydrophobic Clear Acrylic	59.8	59.0	58.4	-
	Hydrophobic Yellow Acrylic	78.0	94.2	96.1	-
Owczarek et al. (16)	Hydrophobic Clear Acrylic	90.6	94.2	95.2	93.7
	Hydrophobic Yellow Acrylic	54.8	88.7	86.8	87.8

No standard has emerged from the literature for reporting overall transmittance. Some publications report transmittance at key wavelengths, and others only overall transmittance. Further compounding the problem is that the method for calculating overall transmittance varies. Some publications do not report their method (e.g., if it is a median or average value, or the wavelength range over which the statistic is calculated). Still others do clarify the method, but problems remain. For example, Werner et al. (18) measure, with 1-nm sampling frequency, the transmittance of IOLs across a 550 nm bandwidth (300–850 nm), but the authors report only a single overall transmittance calculated as an average across in the visible range (400–700 nm): 98.9% for PMMA IOLs. This “overall transmittance" method of reporting one value over a wavelength range is less valuable than reporting the transmittance curve, because there are many possible absorption spectra that could yield an 98.9% average transmittance. The overall transmittance of a material can only be meaningfully relied upon if the shape of the transmittance spectrum is also given. Moreover, almost all reports of transmittance are limited to the visible range, even though many clinical retinal imaging systems function in the near infrared range. IOL fabrication should take into consideration these other imaging techniques that may be necessary at some point for the patient due to other clinical indications. A proposed best practice for those reporting optical transmittance for IOLs is to provide the transmittance values at 5-nm sampling frequency extending from the visible into the near infrared range.

With regard to absolute transmittance levels across the visible range, the PMMA measured by Michelson et al. (19) demonstrated the highest transparency. These tests were consistent with the relative improvement of PMMA over hydrophilic acrylic and silicone in other studies (18, 19). Wang et al. (13) demonstrated an increase in transmittance over PMMA with a POSS-PMMA copolymer. The incorporation of polyhedral oligomeric silsesquioxane (POSS) improved surface hydrophobicity and roughness, leading to enhancement of cell viability and spreading of human lens epithelial cells (HLECs) and improving bio-compatibility. While POSS-PMMA copolymers seem promising, there are limited published studies and no long-term testing results available. Moreover, there is a more recent report of many technical challenges hindering the clinical translation of POSS-PMMA copolymer IOLs and a corresponding proposal for improving anti-biofouling (20). There remains a research gap in the long-term efficacy of POSS-PMMA copolymers for use in implanted IOLs and as a material for 3D printing. Readers may see more on the IOL biomaterial discussion, including POSS-PMMA, in the short review by Khader and Fahoum (21).

## 3 Optical scattering

While most of the visible wavelengths of light passing through the ocular media are transmitted, the scattering properties of the eye play also an important role in vision. Light scattering—the redirection of light due to its dielectric interactions with the media it traverses—greatly affects visual acuity (22). Light may scatter in different specular directions as a function of the light's wavelength and the size of the scatterer (23, 24).

In the cataract lens, scattering accounts for much of the optical disturbance. Sources of scattering include modifications to cytoplasmic crystallin proteins and resulting aggregation (25–29), nuclear fiber cell membrane damage (30, 31), and the presence of multilammelar bodies (MLBs) (32–35).

For artificial IOLs, scattering due to native material imperfections should be minimized. Additionally, IOLs should be designed such that post-implantation scattering (e.g., due to biofouling) is inhibited. In this mini review, we limit the presentation of optical scattering to pre-implantation (“inventory") IOLs and functioning IOLs explanted from cadavers for time-dependent performance. The inventory or cadaver origin is indicated under the heading “Condition" in Table 2. Though many of the cited studies examine failed and subsequently explanted IOLs, the scientific discussion of optical scattering changes in IOLs that have failed is more complex (e.g., snowflake degeneration, calcification within the IOL, calcification on the IOL anterior surface, calcification on the IOL posterior surface, etc.) and beyond the scope of this review.

**Table 2:** A review of optical scattering values for different IOL materials and methods.

**References**	**Material**	**Condition**	**Hydration**	**Surface**	**Scattering [CCT]**
Michelson et al. (19)	PMMA	Inventory	Dry	I Max	9
	Hydrophilic Acrylic	Inventory	Dry	I Max	19 ± 6
	Silicone	Inventory	Dry	I Max	5
Ong et al. (36)	Hydrophobic Acrylic	Cadaver	Unprocessed	A&P Avg	40 ± 22
			Dry	A&P Avg	3 ± 2
			Wetted	A&P Avg	16 ± 10
			Hydrated	A&P Avg	41 ± 19
		Inventory	Unprocessed	A&P Avg	2 ± 1
			Dry	A&P Avg	4 ± 2
			Hydrated	A&P Avg	2 ± 1
Morris et al. (47)	Hydrophobic Acrylic	Cadaver	Hydrated	A Max	43 ± 43
				I Max	31 ± 19
				P Max	43 ± 38
		Inventory	Hydrated	A Max	4 ± 3
				I Max	17 ± 28
				P Max	5 ± 4
	Hydrophilic Acrylic	Cadaver	Hydrated	A Max	12 ± 1
				I Max	19 ± 4
				P Max	10 ± 1
		Inventory	Hydrated	A Max	5
				I Max	13
				P Max	7
	PMMA	Cadaver	Hydrated	A Max	7 ± 3
				I Max	0 ± 1
				P Max	7 ± 5
		Inventory	Hydrated	A Max	5
				I Max	1
				P Max	10
	Silicone	Cadaver	Hydrated	A Max	18 ± 16
				I Max	12 ± 5
				P Max	19 ± 18
		Inventory	Hydrated	A Max	5 ± 4
				I Max	3 ± 6
				P Max	5 ± 2
Ogura et al.(37)	Hydrophobic Acrylic	Cadaver	Dry	A&P Avg	5 ± 4
			Wetted	A&P Avg	25 ± 15
			Hydrated	A&P Avg	104 ± 26
		Inventory	Dry	A&P Avg	6 ± 4
			Wetted	A&P Avg	3 ± 2
			Hydrated	A&P Avg	4 ± 2
Bissen-Miyajima et al. (46)	Hydrophobic Acrylic	*In-Vivo*		A Max	109 ± 16
				I Max	46 ± 10
				P Max	90 ± 20
Werner et al. (14)	Hydrophobic Acrylic	Cadaver	Hydrated	A&P Avg	52 ± 49
		Inventory	Hydrated	A&P Avg	14 ± 4
Barra et al. (69)	Hydrophobic Acrylic	Inventory	Hydrated	A Max	5 ± 3
Hayashi et al. (45)	Hydrophobic Acrylic	*In-Vivo*		A Max	103 ± 27
				I Max	13 ± 10
	Silicone	*In-Vivo*		A Max	7 ± 5
				I Max	2 ± 2
	PMMA	*In-Vivo*		A Max	7 ± 8
				I Max	1 ± 1

A, anterior surface; I, internal matrix; P, posterior surface.

In the analysis of IOL optical scattering, Scheimpflug imaging is used to quantify relative amounts of light scattered from the ocular lens back to a detector aligned anterior to the object along the optical axis. For artificial IOL imaging, results are often expressed in units of computer-compatible tape (CCT, i.e., an 8-bit integer ranging from 0 to 255 where 255 represents high scattering). In papers measuring the scattering properties of IOLs, the presentation of results varies, including scattering from the anterior (A) surface, posterior (P) surface, or internal (I) matrix. The values are reported as a maximum of one surface or an average of multiple surfaces.

In addition to the measurement method, another important consideration is the condition of the tested IOL. Ong et al. (36) demonstrated that the scattering properties of IOLs are highly dependent on if the IOL is dry, wetted (≥ 2 min of hydration), or hydrated (≥ 2 h of hydration). IOLs labeled as “unprocessed" are those that are immediately imaged upon extraction, and IOLs imaged *in-vivo* are labeled accordingly. These notes are included in Table 2. under the heading “Hydration."

From the studies reviewed, a few key observations may be drawn. First, several studies (36, 37) demonstrate that the scattering behavior highly depends on the lenses' hydration condition (dry, wetted, or hydrated) because of so-called glistening or nanoglistening (36). Kato et al. (38) demonstrated that small temperature fluctuations led to micro-vacuole formation, and Saylor et al. (39) proposed a osmotic cavitation mechanism for glistening formation in which pressure differences form small pockets of water. The distribution of glistening ranges in size from 1 to 20 μ*m* (slightly larger than the wavelength of visible light), and their scattering properties may be well described by Mie scattering. As the hydration environment of the IOL varies, it would be expected that the micro-vacuoles eventually fill with the surrounding media, changing the refractive index of the scatterer. According to Mie scattering theory, the measure scattering profile will vary with the refractive index of the scatter changes. Interestingly, glistening is known to occur more often in hydrophopic materials (40, 41).

Nanoglistening is distinguished from glistening by the size of the vacuoles. Nanoglistening vacuoles are smaller than the wavelength of visible light—on the range of 140 to 185 nm (42)—and may best be approximated with Rayleigh scattering theory. Nanoglistening appears as IOL whitening (43, 44) and forms by water aggregating in subsurface volumes (43). This is the main source of surface scattering in IOLs (36).

According to the hydrostatic and osmotic pressure mechanisms of micro- and nano-vacuole formation, it can be reasonably extrapolated that studies conducted *in-vivo* [e.g., Hayashi et al. (45) and Bissen-Miyajima et al. (46)] also have scattering values affected by the IOL's environment and should not be directly compared to the IOLs tested in other environments.

Moreover, the scattering properties of IOLs extracted from cadavers were not similar to those taken from inventory (i.e., control IOLs) (14, 36, 37, 47). These scattering differences were further exaggerated in IOLs extracted from patients due to IOL failure (not included in Table 2) (19). This indicates that calcification or other pathologic processes are negatively impacting scattering properties of IOLs *in-vivo*, even when the impact is either unnoticed or deemed unnecessary to treat by clinicians.

Finally, comparing IOL materials across the reviewed studies, PMMA exhibits the best scattering performance, scattering the least light on average in studies that directly compared PMMA to alternatives. (19, 45, 47). Still, hydrophobic acrylic lenses did not significantly impair optical performance, despite higher scattering levels, as evidenced by similar MTF (modulation transfer function) and Badal image resolution values between hydrated explanted IOLs and controls (47).

## 4 Additive manufacturing and IOLs

Additive manufacturing holds much potential in the area of ophthalmology, with unique possibilities of creating custom optics for patient personalization (48, 49). While there are many possible ophthalmic applications of additive manufacturing, IOLs hold unique promise. Printed materials may be combined with so-called 4D methods, which consider the object's response to its changing environment in time. For example, drug-eluting implants could serve to administer various medications *in-vivo* (50, 51) and in response to physiological conditions (e.g., change in pH or pressure).

Additionally, 3D-printed IOLs may also be able to mimic the refractive index of the ocular lens. The lens' refractive index is not constant through its volume but is a gradient, and the gradient changes with age (52, 53). It has been demonstrated that the lens' gradient refractive index compensates for optical aberrations introduced by the cornea (54). The highest refractive index values are in the nucleus of the lens, and the lowest values in the cortex (55–57). Additive manufacturing techniques have recently been used to create lenses(58, 59), including lenses with a gradient refractive index (60). By combining such efforts with ongoing work in bio-compatible additive manufacturing (61), bio-compatible gradient index lenses could work to optimize visual acuity for cataract patients.

An emerging research area is in IOL production with additive manufacturing techniques. The field is young and in the stage of material selection and method fine-tuning. Hydrogels hold several material properties that make them candidates for 3D-printed IOLs: bio-compatibility, hydrophilicity, optical transparency, and mechanical strength (62–64). Li et al. (65) reported a 3D-printed IOL made of a poly(acrylamide-co-sodium acrylate) hydrogel with promising bio-compatibility. Debellemanière et al. (66) reported a 3D-printed Ridley-style (67) IOL using UV-cured PMMA. The lens achieved an average of 75% transmittance in the visible range, well below the transmittance of IOLs reported in Table 1. Hidalgo-Alvarez et al. (68) reported stereolithographic prototyping of a IOL from a photopolymerizable resin (2-phenoxyethyl acrylate, polly (ethylene glycol) dimethacylate) and included transmittance data, but no scattering data. Furthermore, the scalability and reproducibility of the technique must be demonstrated before adoption. None of the recent studies has sufficiently examined the standard optical properties of the 3D-printed IOLs, focusing instead on bio-compatibility and more isolated measurement techniques for optical characterization. Moreover, issues of long-term viability are underexplored. After developing methods to simulate long-term use, standard optical measurements (scattering and transmittance) may be used to assess optical performance after long-term use.

This mini review has demonstrated, over the past 10 years, optical transmittance and optical scattering have become the standard for IOL characterization, and both techniques should be performed as a standard assessment method for IOL prototyping. Scattering data should include an assessment spectrum extending from the visible through near infrared wavelengths.

We have thus situated emerging additive manufacturing technologies in the recent historical context of IOL efficacy. Researchers working in additive manufacturing for IOL production may demonstrate the efficacy of a 3D-printed IOL in comparison to other viable solutions with standard optical transmittance and optical scattering techniques. Such reporting will enable fair comparison across IOL technologies.

## 5 Discussion

In summary, this mini review has examined two optical properties of IOLs used in cataract surgeries that have become standard measurements for IOL comparison: (1) optical transmittance and (2) optical scattering. We have combined the results of various IOL materials (PMMA, hydrophobic acrylic, hydrophilic acrylic, and silicone) and IOL conditions (inventory, explanted from cadavers, and *in-vivo*) across many studies, drawing out the superior performance of PMMA and PMMA copolymers. Finally, the emerging field of 3D-printed IOLs was reviewed. Additive manufacturing approaches in IOL production are immature but promising. Researchers in this area should understand the recent historical context of optical transmittance and optical scattering as tools for standardized comparisons across types of IOLs.
